# Prognostic Factors in Severe Eosinophilic Asthma in a Pediatric Population: A Prospective Cohort Study in Spain

**DOI:** 10.3390/jcm13237202

**Published:** 2024-11-27

**Authors:** Clara Padró-Casas, María Basagaña, María del Mar Martínez-Colls, Ignasi García-Olivé, Carlos Pollan Guisasola, Aina Teniente-Serra, Eva Martínez-Cáceres, José Tomás Navarro, Carlos Martínez-Rivera

**Affiliations:** 1 Allergy Section, Severe Asthma Unit, Hospital Universitari Germans Trias i Pujol, Faculty of Medicine, Universitat Autònoma de Barcelona (UAB), Carretera de Canyet s/n, 08916 Badalona, Spain; 2 Pediatric Department, Severe Asthma Unit, Hospital Universitari Germans Trias i Pujol, 08916 Badalona, Spain; 3 Pneumology Department, Severe Asthma Unit, Hospital Universitari Germans Trias i Pujol, Faculty of Medicine, Universitat Autònoma de Barcelona (UAB), 08916 Badalona, Spain; 4 Otorhinolaryngology Department, Severe Asthma Unit, Hospital Universitari Germans Trias i Pujol, 08916 Badalona, Spain; 5 Immunology Department, Severe Asthma Unit, Hospital Universitari Germans Trias i Pujol, Faculty of Medicine, Universitat Autònoma de Barcelona (UAB), 08916 Badalona, Spain; 6 Department of Hematology, Hospital Universitari Germans Trias i Pujol, Universitat Autònoma de Barcelona (UAB), Institut Català d’Oncologia, Josep Carreras Leukaemia Research Institute, 08916 Badalona, Spain

**Keywords:** eosinophilic asthma, severe asthma, pediatric asthma, induced sputum, biomarkers, cohort study

## Abstract

**Background/Objectives**: The objective of this study was to provide real-world data on prognostic factors in children with severe eosinophilic asthma and to assess biomarkers of outcome. **Methods**: Fifty-nine children (aged 6–17 years) were included in a prospective cohort attended in a Severe Asthma Unit of a tertiary care teaching hospital in Badalona (Barcelona, Spain) and visited at baseline and at 1-year follow-up. Study variables included asthma control using the Asthma Control Test (ACT), forced expiratory volume in one second (FEV_1_), exacerbation episodes, fractional exhaled nitric oxide (FeNO), and inflammatory biomarkers (blood tests, sputum cells, immunoallergic tests, and levels of cytokines and effector cells in blood and sputum). **Results**: There were 36 boys and 23 girls, with a mean (SD) age of 11.9 (2.8) years. Uncontrolled severe asthma was diagnosed in 83.1% of cases, with poor symptom control (ACT score < 20) in 52.5%, obstructive pattern (FEV_1_ < 80% predicted) in 35.6%, and more than one exacerbation in the previous year in 30.5%. The mean duration of asthma was 9.2 (3.6) years. Positive prick tests were recorded in 55 patients, with polysensitization in 6. The mean percentage of sputum eosinophils was 2.5% (3.1%), and the mean eosinophil blood count 543.4 (427.7) cells/µL. Ten patients (32%) showed sputum eosinophilia (>3% eosinophils). Sputum eosinophils did not correlate with blood eosinophils, FeNO, and serum periostin. At 12 months, 13 (22%) children had uncontrolled asthma and 46 (78%) had controlled asthma. Variables significantly associated with uncontrolled asthma were duration of asthma (OR = 1.23, 95% CI 1.01–1.49, *p* = 0.04) and an ACT score < 20 (OR = 0.80, 95% CI 0.69–0.93, *p* = 0.004). Lower serum levels of IL-9 appeared to be related with uncontrolled asthma, but statistical significance was not reached. **Conclusions**: Pediatric severe eosinophilic asthma showed a predominant allergic phenotype with symptomatic disease as a main contributor of uncontrolled asthma at 1 year. Predictive biomarkers of outcome were not identified. Further studies are needed to confirm the present findings especially considering additional variables for a better phenotypic characterization of severe eosinophilic asthma in children and to study in-depth the role of inflammatory biomarkers.

## 1. Introduction

Asthma is the most common chronic lung disease in childhood [1] and is among the 20 most prevalent conditions worldwide in terms of disability-adjusted life years in the pediatric population [2]. The prevalence of the disease varies largely between 15.1% and 51.1% according to different definitions of childhood asthma [3] especially due to variable presenting manifestations of asthma in children and consequently clear divergences among investigators in the definition of asthma [1]. A combination of genetic and environmental factors is involved in the appearance of asthma, including atopy, prematurity, smoking habit of the mother, viral infection during childhood (such as rhinovirus), or exposure to environmental pollution [4]. Although most children with asthma achieve good symptom control with standard therapies, severe asthma represents approximately 5% of cases but accounts for nearly 50% of all asthma-related expenditures [5]. Moreover, around one in four asthmatic children with severe disease had difficult-to-control asthma [6] in which the asthma is uncontrolled despite optimal management. Poor adherence to controller medications, insufficient avoidance of asthma triggers, or inadequate treatment of comorbidities are some common factors contributing to uncontrolled asthma [7]. In fact, uncontrolled asthma continues to be a challenge for clinicians since these patients remain at an increased risk of developing severe exacerbations and persistent airflow limitations, have a poor quality of life, and require multidisciplinary long-term management strategies [8].

Interest in the heterogeneity of clinical phenotypes-endotypes and the biological mechanisms underlying severe pediatric asthma has substantially increased in recent years [9,10]. Allergic asthma is the most prevalent phenotype in children, characterized by allergic sensitization, atopic comorbidities, and a Th2-high inflammatory profile with high IgE serum levels, eosinophilia, and elevated fractional exhaled nitric oxide (FeNO) [4]. The phenotype of severe non-allergic eosinophilic asthma in the pediatric population is rare [11] and there is a lack of research aimed to assess the phenotype of severe asthma in children, which in fact was the purpose of the present study. To date, the percentage of eosinophils in sputum has been the best characterized biomarker in asthmatic patients [12]. In children, like adults, there is an increase in sputum eosinophilia in symptomatic periods and during exacerbations [13,14] in association with bronchial hyperreactivity and airway obstruction [15], although recent studies have shown instability of sputum eosinophil counts in pediatric asthma populations [16]. However, obtaining induced sputum is not always feasible in many centers. Peripheral eosinophilia is an easier approach to assess the phenotype of severe asthma, and a good correlation between sputum and blood eosinophilia has been reported in adults [17]. On the contrary, scientific evidence is limited in children and it has been shown that blood eosinophil counts rarely reflect airway eosinophilia [18]. We believe that this is an interesting line of research to be further explored.

On the other hand, serum levels of periostin have been recognized as a biomarker of Th2-driven inflammatory responses, but its usefulness in childhood asthma has not been established [19]. Therefore, we believe that its correlation with other T2 inflammatory markers should be determined. Moreover, Th2 type cytokines are difficult to detect in children and their levels have shown great variability among patients [20], and this is probably one of the reasons for which the immunological profile and its relationship with control of asthma in children with severe eosinophil asthma has not been extensively examined. Recently, it has been also shown that innate immunity mediated by ILC2 lymphoid cells and not only adaptive immunity has a prominent role in the pathophysiology of eosinophilic asthma [21], so we consider it necessary to study the inflammatory profile and effector cells in children with severe eosinophilic asthma.

In order to contribute to a better clinical characterization of children with severe eosinophilic asthma, the present prospective cohort study was designed to assess the correlation between sputum eosinophils and other biomarkers such as FeNO, blood eosinophil count, and serum periostin levels, as well as to identify prognostic biomarkers of asthma control after 12 months of follow-up. These findings would contribute to establishing a more effective and personalized therapeutic approach.

## 2. Materials and Methods

### 2.1. Study Design and Participants

This was a prospective cohort study carried out in a pediatric asthma population attended at the Severe Asthma Unit of an acute tertiary care teaching hospital in Badalona, Barcelona, Spain. Patients were recruited in successive visits in the Severe Asthma Unit upon meeting the inclusion criteria. Inclusion criteria were age between 6 and 17 years, diagnosis of severe asthma established at least 1 year prior to inclusion in the cohort according to guidelines of the Global Initiative for Asthma (GINA) [22], peripheral eosinophil blood count of ≥220 cells/µL [17], and provision of written informed consent. It was also required that exclusion criteria were the presence of immunological, hepatic, renal, or heart diseases that according to the investigator’s criteria may interfere with the execution of the study, clinically relevant upper respiratory tract abnormalities, severe psychiatric disorders, any condition preventing cooperation with the study procedures, impossibility to attend the study visits, poor adherence to asthma treatment, and refusal to participate. Severe asthma was defined as disease that remains uncontrolled despite treatment with high doses of inhaled corticosteroids (ICS)-long-acting beta2-agonists (LABAs) (ICS-LABA) or required high-dose ICS-LABA for preventing the disease from becoming uncontrolled [1]. After inclusion in the study, all patients were followed for 1 year.

The study was carried out following the principles of the Declaration of Helsinki, classified as a non-interventional post-authorization observational study by the Spanish Agency for Medicines and Medical Devices (AEMPS), and approved by the Clinical Research Ethics Committee of Hospital Universitari Germans Trias i Pujol (Badalona, Barcelona, Spain) (code PI-15-076, approval date 10 July 2015). Written informed consent was obtained from all participants and their parents or legal guardians using specific informed consent versions for children < 12 years and for those 12–17 years.

### 2.2. Study Procedures

The study was conducted between September 2015 and February 2018 and included a baseline visit (visit 0) and three consecutive visits (visits 1 to 3) at 4-month intervals over the 1-year follow-up period. At each of the four study visits (baseline and follow-up), the following tests/procedures were carried out: standardized medical history, pulmonary function tests, FeNO measurement, degree of asthma control, and asthma quality of life. At the baseline visit (visit 0), all patients underwent a prick test, measurement of serum levels of total IgE and specific IgE, sputum cellularity, sputum and serum levels of interleukins (IL) IL-4, IL-5, IL-8, IL-9, IL-13, and IL-17, serum periostin, and quantification of Th1, Th2, Th17, ILC1, ILC2, and ILC3 cell populations.

FeNO (Niox®, Oxford, UK) was measured following recommendations of the European Respiratory Society and the American Thoracic Society (ERS/ATS) [23]. The degree of asthma control was evaluated using the Asthma Control Test (ACT) for children aged ≥12 years [24] or the Childhood Asthma Control Test (c-ACT) for those aged <12 years [25] (an ACT or c-ACT score ≥ 20 indicates well-controlled asthma). Asthma quality of life was measured with the Asthma Quality of Life Questionnaire (AQLQ) for children aged ≥12 years [26] (scores range 1–7 with higher scores indicating better quality of life) or the 23-item Pediatric Asthma Quality of Life Questionnaire (PAQLQ) for those aged <12 years [27] (the overall score is the mean of all 23 responses and the 3 domain scores of symptoms, activity limitation, and emotional function are the means of the items in those domains, with higher scores indicating better quality of life). Spanish validated versions of these instruments were used.

The definition of uncontrolled asthma required the presence of at least one of the following characteristics: (1) poor control of symptoms with an ACT or c-ACT score < 20; (2) severe exacerbation episodes requiring treatment with two or more courses of systemic corticosteroids for more than 3 days in each episode since the last visit; (3) severe exacerbations requiring at least one admission to the hospital, stay in an intensive care unit (ICU) or mechanical ventilation since the last visit; and (4) airflow limitation with forced expiratory volume in one second (FEV1) < 80% predicted after bronchodilation with a short-acting beta agonist. Prick tests (commercial extracts supplied by LETI Pharma and Roxall Medicina España, S.A.U, Zamudio, Spain) were performed with a battery of standard allergens [28] to assess the immunoallergic status, which was completed with a measurement of serum levels of total IgE and specific IgE according to sensitizations identified using ImmunoCAP™ (Thermo Fisher Scientific, Walthma, MA, USA) when patients were sensitized to <3 allergen families or ImmunoCAP™-ISAC assay (Thermo Fisher Scientific, Walthma, MA, USA) for allergen component testing in polysensitized patients (≥3 allergen families). Results of immunoallergic testing were evaluated independently by two experienced allergologists in order to identify clinically relevant sensitizations. Patients were also visited by an otorhinolaryngologist to assess the presence of nasal polyposis or other anatomical abnormalities (using a flexible Olympus^®^ rhinolaryngoscope (Shinjuku City, Tokyo)).

Sputum samples were obtained using the induced sputum technique (Ultrasonic Nebuliser Model NE-U17, Omron Electronics Iberia S.A.U., Madrid, Spain) based on inhalation of increasing concentrations (3%, 4%, 5%) of hypertonic saline as described by Pizzichini et al. [29] and Djukanovic et al. [30] and adapted according to the manual of procedures of the Spanish Society of Pneumology and Thoracic Surgery (SEPAR) [31]. The sputum cellularity was evaluated by microscopic examination after staining with May-Grünwald-Giemsa and expressed as cell percentage. Induced sputum viability was defined as <15% epithelial cells. Sputum cellularity was used for the definition of four patient profiles including a neutrophilic profile (eosinophils < 3%/neutrophils > 61%), an eosinophilic profile (eosinophils > 3%/neutrophils < 61%), a mixed granulocytic profile (eosinophils > 3%/neutrophils > 61%), and a pauci-granulocytic profile (eosinophils < 3%/neutrophils < 61%). Also, IL-4, IL-5, IL-8, IL-9, IL-13, and IL-17) were measured in induced sputum samples using the Cytometric Bead Array (CBA) system (BD Biosciences, San Jose, CA, USA), with results expressed as pg/mL. A criterion of <40% cell death was established as induced sputum sample viability for IL analysis. Serum levels of IL-4, IL-5, IL-8, IL-9, IL-13, and IL-17 measured with the CBA system, and serum periostin levels using a Periostin Human ELISA kit (Thermo Fisher Scientific, Walthma, MA, USA). Results of IL and periostin were expressed as pg/mL and ng/mL, respectively. Finally, flow cytometry was used for quantification of Th1, Th2, Th17, ILC1, ILC2, and ILC3 cell populations in fresh blood samples.

### 2.3. Study Outcomes

The outcomes of the study were as follows:

- To characterize a population of children with severe eosinophilic asthma from a clinical perspective and asthma biomarkers.
- To assess the correlation between sputum eosinophils and other biomarkers, such as blood eosinophil count, FeNO, and serum periostin levels.
- To identify prognostic markers of asthma control after 12 months of follow-up.

### 2.4. Statistical Analysis

Categorical variables are expressed as frequencies and percentages, and continuous variables as mean and standard deviation (SD). The distribution of variables departed from normality according to the Kolmogorov–Smirnov test. The Mann–Whitney *U* test was used for the comparison of quantitative variables between the groups of controlled and uncontrolled asthma over the 12 months of follow-up. The Spearman rank correlation coefficient (R) was used to assess the relationship between sputum eosinophils and serum periostin levels, FeNO, and peripheral blood eosinophil count. The sensitivity, specificity, and predictive values of different thresholds of peripheral eosinophils (220 and 300 cells/µL) and FeNO (20 and 40 ppb) for predicting sputum eosinophils > 3% were calculated. The level of agreement between the two allergologists in the identification of clinically relevant allergic asthma was evaluated with the kappa statistic.

A logistic regression model with backward stepwise selection was used to assess factors associated with uncontrolled asthma at follow-up. Clinically relevant variables with a *p* < 0.2 in the bivariate analysis were included in the model as the independent variables, with uncontrolled asthma at 12 months of follow-up as the dependent variable. Odds ratio (OR), 95% confidence intervals (CIs), and the area under the ROC curve (AUC) for the regression model were calculated. Statistical significance was set at *p* < 0.05. The Statistical Package for the Social Sciences (SPSS) version 22.0 (IBM Corporate, Armonk, NY, USA) was used for the analysis of data.

Additionally, in order to identify hidden factors potentially associated with bad control of asthma, an automated stratified exploratory data analysis process (AutoDiscovery v. 07/09/2021, Butler Scientifics, Barcelona, Spain) was performed. This process was carried out in each of the possible subgroups of the data set generated by means of a list of previously selected stratification factors. Subgroups or associations with a sample size less than 5, a sample size less than 1% of the total sample size, or a significance level α (two-sided test) equal to or greater than 0.05 were automatically rejected. Finally, an expert assessment of the registered results was carried out to select the most relevant results related to the original goals.

## 3. Results

### 3.1. Characteristics of the Study Population

The study population included 59 children (36 males, 23 females) with a mean (SD) age of 11.9 (2.8) years (range 6–17). Uncontrolled severe asthma was diagnosed in 49 children (83.1%), with poor symptom control (ACT score < 20) in 31 (52.5%), obstructive pattern (FEV_1_ < 80% predicted) in 21 (35.6%), and more than one exacerbation in the previous year in 18 (30.5%). The main findings at the initial study visit are shown in Table 1.

In 56 children (94.9%), allergic sensitization studies were performed, with positive prick testing in 55 (98.2%) and polysensitization to allergens in 6 (10.7%). Specific IgE was measured in 54 children. As shown in Table 2, sensitization to dust mites and pet dander were the most common. In 42 of 59 children (71.2%), the two independent allergologists considered that they had clinically relevant allergic asthma (kappa correlation coefficient 0.761).

### 3.2. Sputum and Peripheral Blood Eosinophils

The induced sputum technique was performed on the 59 patients, but it was necessary to stop or modify the procedure in 7 (11.9%) due to coughing or nausea. Samples were obtained in 35 of the 59 procedures (59.3%). Cell counts could be performed on 31 of the 35 samples (88.6%), but ILs were only assessable in 8 cases (22.9%).

The mean percentage of eosinophils in induced sputum was 2.5% (3.1%) and the mean eosinophil blood count was 543.4 (427.7) cells/µL. Sputum eosinophilia (>3% eosinophils) was found in 10 patients (32.3%). The mean percentage of sputum neutrophils was 51.4% (32.5%) and the percentage of patients with >61% sputum neutrophils was 45.2%. Based on sputum cellularity, 9 (29.0%) patients showed an eosinophilic profile, 7 (22.6%) a neutrophilic profile, 7 (22.6%) showed a mixed profile, and 8 (25.8%) showed a pauci-granulocytic profile.

A correlation between sputum eosinophils and FeNO values, peripheral eosinophil count, and serum periostin levels was not found (Table S1 of the Supplementary Materials). In the analysis of different thresholds of peripheral blood eosinophil count (220 and 300 cells/µL) and FeNO (20 and 40 ppb) for predicting sputum eosinophilia > 3% did not show significant results. The eosinophil count of 300 cells/µL was the cutoff value associated with the best sensitivity (60%), specificity (38.1%), and positive and negative predictive values (31.6% and 66.7%, respectively). The FeNO of 20 ppb showed a sensitivity of 50%, specificity of 33%, and positive and negative predictive values of 29.4% and 54.5%, respectively.

### 3.3. Inflammatory and Immune-Related Variables

Mean (SD) values of FeNO and serum periostin were 46.8 (49.0) ppb and 11,858 (12,310) pg/mL, respectively. Detailed data of sputum and blood ILs and cell populations of the innate and adaptive immune pathways are shown in Table 3.

### 3.4. Asthma Control at 12 Months of Follow-Up

During the 12-month follow-up period, there were 13 patients (22.0%) in whom asthma was never controlled and 46 (78.0%) with controlled asthma, including persistently (always) controlled asthma in 12 (20.3%) and occasionally controlled asthma in 34 (57.6%). The distribution of clinical variables, allergic sensitization, sputum and peripheral blood eosinophils, sputum cellularity profiles, total IgE, FeNO, and serum periostin levels were similar in the groups of children with never controlled asthma and sometimes/always controlled asthma, except for a significantly higher duration of asthma and symptomatic patients (ACT score < 20) in the group of uncontrolled asthma, with exacerbations in the previous year and use of systemic corticosteroids also being more frequent and marginally statistically significant. Patients with never controlled asthma at follow-up also showed a significantly lower ACT score at the initial visit (Table 4).

Results of inflammatory parameters of innate and adaptive immunity did not show significant differences between the groups of children with never controlled and always/sometimes controlled asthma, although mean serum levels of IL-9 were higher in controlled asthma (3.9 vs. 2.14 pg/mL; *p* = 0.069) and NCR^+^ILC3 were higher in uncontrolled asthma (0.01 vs. 0.004‰; *p* = 0.090). Data of inflammatory and immune-related variables in sputum and peripheral blood at 12 months of follow-up are shown in Table S2 of the Supplementary Materials.

**Figure 1:**
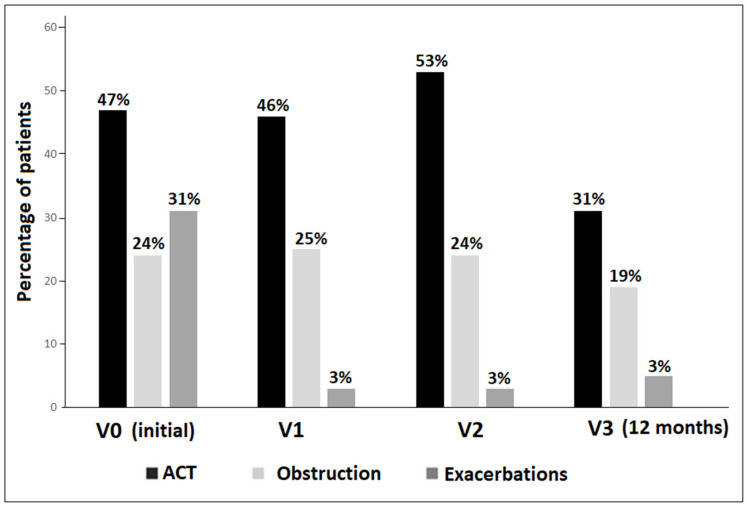
Percentages of patients with uncontrolled asthma during the study period considering the criteria of ACT score < 20, airflow obstructive pattern (<80% predicted), and exacerbation episodes separately (V0: initial visit, V1: study visit at 4 months; V2: study visit at 8 months; V3: study visit at the end of the 12-month follow up period; exacerbation episodes in the last year were only considered at V0).

When the criteria of an ACT score < 20, airflow obstructive pattern (FEV_1_ < 80% predicted), and number of exacerbations was considered, inadequate symptom control was the predominant feature over the 12-month follow-up period followed by airflow obstruction and exacerbation episodes (Figure 1).

In the multivariate analysis, the only two variables significantly associated with uncontrolled asthma at 12 months were duration of asthma (OR = 1.23, 95% CI 1.01–1.49, *p* = 0.04) and an ACT score < 20 (OR = 0.80, 95% CI 0.69–0.93, *p* = 0.004). Serum levels of IL-9 appeared to be related to uncontrolled asthma, although statistical significance was not reached (OR = 0.83, 95% CI 0.63–1.09; *p* = 0.178). The model showed an AUC of 0.827 (95% CI 0.72–0.93; *p* < 0.000) (Table S3 and Figure S1 of the Supplementary Materials).

**Figure 2:**
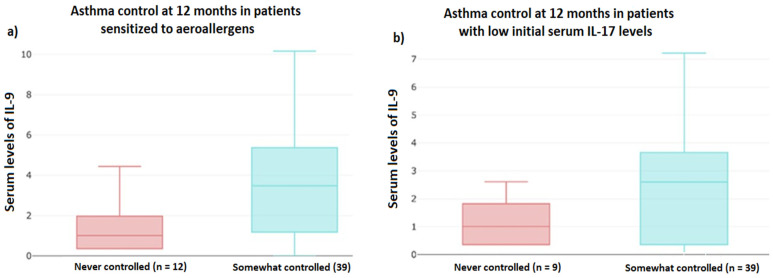
Boxplot of the relationship between serum levels of IL-9 and control of asthma at 12 months: (**a**) in patients sensitized to aeroallergens (left) and (**b**) in patients with initial IL-17 < 1.06 pg/mL.

Finally, the automated stratified exploratory data analysis showed higher serum levels of IL-9 in children with controlled asthma at 12 months, both in the subsets of patients with sensitization to aeroallergens and in the subset of patients with initial IL-17 levels < 1.06 pg/mL (Figure 2). It was also observed that the NCR^+^ILC3 cell population was greater in the subgroup of uncontrolled children without sensitization to tree or *Parietaria* pollens, and that patients with a higher BMI and poorer asthma control were those over the age of 12 (Figures S2 and S3 of the Supplementary Materials).

## 4. Discussion

This prospective cohort study contributes to characterizing severe eosinophilic asthma in children, providing real-world data of demographic and clinical features, allergen sensitization, sputum and serum biomarkers, and innate and adaptive immunity factors including Th1 and Th17 cell populations. A correlation between sputum and blood eosinophilia was not found. Clinical and/or immunological factors that may predict poor control of asthma at 1 year of follow-up were evaluated, and an ACT score < 20 and longer duration of asthma were the only two predictors of outcome.

### 4.1. Characteristics of Patients

Salient features of the cohort included a high percentage of boys (61%), mean age of 12 years, diagnosis of asthma established before the age of 11, and symptomatic disease (ACT score < 20) as the main cause of poor control of asthma at the initial visit. This is in contrast to severe eosinophilic asthma in adults in which impairment of lung function has a prominent role in keeping asthma uncontrolled [32]. The percentage of males exceeding females has been also reported in 188 children of the Severe Asthma Research Program (SARP) III cohort [33] and in a cross-sectional analysis of 8196 children from the Mayo Clinic Olmsted County Birth Cohort [34]. In a comparison of clinical characteristics of adult-onset asthma and childhood asthma, it has been suggested that small airways of children may be more fragile and sensitive to airway inflammation, implying a more severe phenotype with poorer long function and more severe disease in asthma that has persisted since childhood [35]. Our patients suffered from severe asthma and were on treatment according to GINA steps 4–5 and LABA (ICS-LABA). The role of other treatments is irrelevant (a few patients received anti-leukotrienes) and because the study was conducted between 2015 and 2018, most of the biological drugs currently used for the treatment of asthma were not yet available.

We also found a high rate of allergen sensitization (more than 90%), which was clinically relevant in 71% of children. It is known that early-life exposures and sensitization to various allergens occur in children with severe asthma, and the association between allergy and asthma severity is stronger in children [36,37]. Dust mites and pet dander are some of the most common allergens. In a prospective cohort of 300 asthmatic children ages 4–12 followed for 1 year, sensitization of Der p 1 house dust mite allergen and pet allergens were associated with asthma severity, but Der p 1 remained an independent risk factor after accounting for pet allergens and regardless of Der p 1 specific IgE status [38]. In the Multicenter Allergy Study cohort, a birth cohort started in 1990 in Germany, the number of house dust mite-component specific sensitizations increased with disease severity and with age, and sensitization to Der p 1 and Der p 23 before the age of 5 years was predictive of asthma at school age [39].

### 4.2. Inflammatory and Immune-Related Parameters

Induced sputum is a safe technique in pediatric asthma. Samples were obtained in 35 of the 59 procedures, with a 59.3% effectiveness, which is similar to 60% reported in other series of pediatric asthma [40]. Sputum cellularity was analyzed in 88.6% of cases, and the mean eosinophil value of 2.5% and the rate of children with sputum eosinophilia > 3% of 32.3% were lower than expected despite the inclusion criterion of a peripheral eosinophil blood count of ≥220 cells/µL. These findings may be explained by chronic treatment with high-dose inhaled corticosteroids and the fact that sputum sampling was performed during exacerbation-free periods [12]. However, the mean blood eosinophil count of 543.4 cells/µL was high. This discrepancy between sputum and blood eosinophils was also observed in the children asthma population of the Severe Asthma Research Program (SARP) III cohort [33]. The low correlation between sputum and blood eosinophils may suggest the presence of differential tissue-dependent cell dynamics, which would be more marked in children. A neutrophilic profile was found in 22.6% of participants, although sputum neutrophils do not represent established markers to define the non-type 2 asthma endotype in children [11]. The lack of correlation between sputum and blood eosinophils suggests that other clinical variables should be considered when phenotyping pediatric populations with severe asthma.

Sputum eosinophilia was not correlated with other biomarkers, including peripheral blood eosinophilia, FeNO, and serum periostin, a finding that has been also reported in other studies of pediatric asthma [41,42,43]. A cut-point of 300 cells/µL of blood eosinophils showed a 60% sensitivity and 38.1% specificity for estimating sputum eosinophilia. Although studies in adult-onset asthma have shown a good correlation between these two biomarkers [32,44], our results indicate a poor correlation between T2 biomarkers and sputum eosinophilia in children, so that findings from adults cannot be translated to pediatric asthma populations [42]. Also, we found low levels of ILs even using the CAB system (an accurate and high-cost analytical technique), suggesting that the usefulness of sputum and blood ILs analysis is limited in children with severe eosinophilic asthma, which is characterized by remodeling without Th2 cytokines [19].

### 4.3. Asthma Control at Follow-Up

After 1 year of inclusion in the prospective cohort, children were grouped into those whose asthma was never controlled at none of the study visits and those with controlled asthma at any time over the follow-up period. Control of symptoms (ACT score), pulmonary function, and exacerbations were criteria for the definition of asthma control. In this respect, bronchial obstruction was the main cause of uncontrolled asthma in the cohort of adult asthma patients [32], in contrast to persistence of symptoms in children. Accordingly, a higher percentage of patients with an obstructive respiratory pattern (<80% predicted) among asthmatic adults may account for the differences of uncontrolled asthma between the two cohorts (49% vs. 22%).

In relation to the clinical relevance of asthma exacerbations episodes, the percentage of exacerbations was higher at the initial visit (31%) and decreased to 3% at follow-up. This difference may be explained by the use of different criteria for the assessment of exacerbations at the initial visit (exacerbations in the previous year) and during the study (two or more courses of systemic corticosteroids, each lasting at least 3 days, or in-patient care). It is possible that exacerbations may be underestimated by the definition based on the use of systemic steroids. However, there is a large heterogeneity in the use of oral corticosteroids in asthmatic children. In a study of 212,060 asthma patients 5–17 years old identified in six European electronic databases from the Netherlands, Italy, the UK, Denmark, and Spain in 2008–2013, the use of systemic corticosteroids ranged between 24.7% to 99.9%, with 77% in the UK cohort with the largest number of children (124,554) [45].

In the bivariate analysis, longer duration of asthma, lower mean ACT score, higher percentage of symptomatic patients (ACT score < 20), and use of systemic corticosteroids were significant factors associated with uncontrolled asthma. The profile of allergic sensitization was unrelated to the level of asthma control, and we were unable to identify a molecular biomarker of asthma outcome. Serum levels of IL-9 were higher in controlled asthma, but the NCR^+^ILC3 cell population was higher in uncontrolled asthma, although differences were not significant. Higher levels of NCR^+^ILC3 were also found in our cohort of adults with severe eosinophilic asthma [32]. However, in children, the immune response in severe eosinophilic asthma remains unclear. Automated stratified exploratory data analysis also showed higher IL-9 values in controlled asthma in the subsets of allergen-sensitized patients and in those with low IL-17 values and higher NCR^+^ILC3 in uncontrolled asthma in children without allergen sensitization (tree/weed pollen). These data suggest that individuals with severe eosinophilic asthma who present with a mixed inflammatory profile, characterized by both T2 inflammation and innate immunity with an ILC3 cellular profile, may experience poorer control. This observation may have implications for the potential of therapeutic interventions, such as biological therapies, to effectively manage this patient subgroup. These findings are not conclusive and require further investigation. Finally, we observed that a higher BMI was associated with uncontrolled asthma in patients aged over 12 years, likely reflecting those with more advanced stages of asthma.

In the multivariate analysis, duration of asthma and ACT score < 20 were the only two variables independently associated with uncontrolled asthma. Symptom-based tools can be used as part of the routine evaluation of asthmatic children, although the predictive utility of ACT to assess the risk of asthma exacerbations has been questioned as exacerbations can also occur in the context of reasonable asthma control [46]. However, based on these present results, early introduction of targeted therapy (biologics) may be considered in children who already show poor control according to the ACT score, rather than waiting for other manifestations such as exacerbations or persistent bronchial obstruction.

### 4.4. Limitations and Strengths of the Study

The present findings should be interpreted considering the limitations of the study, such as the single-center design, the reduced sample size, the heterogeneity of the controlled asthma group, and the differences in the number of patients in the categories of uncontrolled and controlled asthma at 12 months. Also, the influence of comorbidities and the variability of biomarkers during the follow-up period were not evaluated. It may be argued that the inclusion criterion of a blood eosinophil count of ≥220 cells/µL was quite a low threshold for eosinophilic asthma, but we considered the characteristics of severe asthma patients chronically treated with medium-to-high doses of corticosteroids, which are known to reduce the baseline blood eosinophil count. All patients had their treatments optimized with good adherence to medication, but the study was conducted before the introduction of biologic agents, which could certainly have improved the control of symptoms. However, the prospective design and the follow-up of over 1-year period are strengths of the study. Moreover, data obtained from a clinical series of children diagnosed with severe eosinophilic asthma and attended in a multidisciplinary Severe Asthma Unit by the same medical team adds evidence of the spectrum of childhood asthma in the real-world setting.

## 5. Conclusions

In the present study, pediatric severe eosinophilic asthma showed a predominant allergic phenotype, and there was no correlation between sputum eosinophils and blood eosinophilia, FeNO, and serum periostin levels, which may question the usefulness of induced sputum in children. Predictive biomarkers of asthma outcome were not identified, although mixed phenotypes with activation of IL-17 pathways through ILC3 seemed to be associated with a worse prognosis. Longer duration of asthma and an ACT score < 20 at the initial visit were the only two independent factors significantly associated with uncontrolled asthma at 1 year of follow-up. Therefore, accurate assessment of symptom control is a crucial component in the effective management of childhood eosinophilic asthma. It is important to emphasize that inconclusive immunological findings require continuing a line of research to clarify the role that a specific immunological profile may have in severe eosinophilic asthma in children, as it will allow for more a personalized management and treatment of the disease.

## Figures and Tables

**Table 1:** Characteristics of children with severe eosinophilic asthma at the initial visit.

Variables	Patients (%)	Mean (SD)
Total patients	59 (100)	
Sex, *n* = 59		
Male	36 (61)	
Female	23 (39)	
Age, years		11.9 (2.8)
Body mass index, BMI, kg/m^2^		21 (4.5)
Obesity, BMI > 30 kg/m^2^, *n* = 59	2 (3.4)	
Duration of asthma, years		9.2 (3.6)
Age at the onset of asthma, *n* = 59		
<11 years	57 (96.6)	
12 to 17 years	2 (3.4)	
Family history of atopy, *n* = 56	33 (58.9)	
Family history of asthma, *n* = 59	30 (50.8)	
Chronic rhinosinusitis, *n* = 51	18 (35.3)	
Inhaled corticosteroids, µg budesonide equivalent		1086 (426)
Systemic corticosteroids, *n* = 59	1 (1.7)	
FEV_1_, L		2.4 (0.9)
FEV_1_, %		87.5 (18.5)
ACT score		18.8 (4.7)
AQLQ score		5.2 (1.2)
Symptomatic patients (ACT > 20), *n* = 59	31 (52.5)	
Patients with an obstructive pattern (FEV_1_ < 80%), *n* = 59	21 (35.5)	
Exacerbations in the previous year		1.24 (1.5)
≥1 exacerbation, *n* = 59	18 (30.5)	
Uncontrolled severe asthma, *n* = 59	49 (83.1)	

SD: standard deviation; FEV_1_: forced expiratory volume in one second; ACT: Asthma Control Test; AQLQ: Asthma Quality of Life Questionnaire.

**Table 2:** Allergens identified in 54 sensitized patients.

Allergen	Sensitized Patients *n* (%)	Specific IgE Mean, kU/L
*Dermatophagoides pteronyssinus*	41 (75.9)	50.15
*Dermatophagoides farinae*	38 (70.4)	44.86
*Acarus siro*	10 (18.5)	11.16
*Lepidoglyphus destructor*	15 (27.8)	5.67
*Aspergillus*	7 (13.0)	3.42
*Alternaria*	7 (13.0)	4.96
*Penicillum*	1 (1.8)	0.03
Dog dander	13 (24.1)	15.99
Cat dander	15 (27.8)	12.87
Horse dander	1 (1.8)	5.32
Olive pollen	10 (18.5)	13.35
Plane tree pollen	4 (7.4)	5.0
Cypress pollen	4 (7.4)	5.95
Parietaria pollen	4 (7.4)	1.24
Mugworth pollen	2 (3.7)	2.01
Chenopodium pollen	1 (1.8)	0.08
Grass pollen	3 (5.6)	2.1
Cynodon pollen	3 (5.6)	1.02

**Table 3:** Inflammatory and immune-related variables in sputum and peripheral blood.

Variables	Mean (SD)
Fractional exhaled nitric oxide (FeNO), ppb	46.8 (49.0)
Serum periostin, pg/mL	11,858.2 (12,310)
Total IgE, kU/L	706.6 (937.1)
Sputum interleukins (IL), pg/mL	
IL-4	7.11 (8.4)
IL-5	2.96 (3.16)
IL-8	4.22 (2.57)
IL-9	10.21 (7.27)
IL-13	5.98 (5.04)
IL-17	24.20 (18.83)
Blood interleukins (IL), pg/mL	
IL-4	2.95 (4.5)
IL-5	1.23 (2.3)
IL-8	14.03 (14.47)
IL-9	3.54 (4.07)
IL-13	0.5 (0.97)
IL-17	1.62 (1.45)
Cell populations	
Th1 effector, %	6.67 (4.12)
Th1 central memory, %	8.42 (2.3)
Th2 effector, %	2.59 (1.94)
Th2 central memory	5.94 (2.3)
Th17 effector, %	1.77 (0.82)
Th17 central memory, %	4.32 (1.87)
ILC1, ‰	0.17 (0.11)
ILC2, ‰	0.51 (0.27)
NCR^−^ILC3, ‰	0.23 (0.14)
NCR^+^ILC3, ‰	0.0052 (0.0058)

SD: standard deviation; NCR: natural cytotoxic receptor.

**Table 4:** Distribution of the study variables at 12 months of follow-up according to the level of asthma control.

Variables	Never Controlled Asthma (*n* = 13)	Sometimes/Always Controlled Asthma (*n* = 46)	*p* Value
Females, n (%)	6 (46.2)	17 (37)	0.490
Age, years, mean (SD)	12.3(2.8)	11.7(2.9)	0.548
Body mass index, BMI, kg/m^2^, mean (SD)	23.09 (4.97)	20.47 (4.21)	0.073
Obesity, BMI > 30 kg/m^2^, n (%)	1 (7.7)	1 (2.2)	0.332
Duration of asthma, years, mean (SD)	11.12 (2.79)	8.70 (3.67)	0.032
Age at the onset of asthma			
<11 years	13 (100)	44 (95.6)	0.444
12 to 17 years	0	2 (4.3)	
Family history of atopy, *n* (%)	9 (69.2)	24 (52.2)	0.389
Family history of asthma, *n* (%)	6 (46.1)	24 (52.2)	0.701
Chronic rhinosinusitis, *n* (%)	4 (30.8)	14 (30.4)	0.871
Inhaled corticosteroids, µg budesonide equivalent, mean (SD)	1169.2 (415.1)	1062.8 (431.8)	0.346
Systemic corticosteroids, *n* (%)	1 (7.7)	0	0.058
FEV_1_, L, mean (SD) (V0)	2.21 (0.63)	2.5 (0.91)	0.481
FEV_1_, %, mean (SD) (V0)	80.77 (13)	89.43 (19.51)	0.185
ACT score, mean (SD) (V0)	15.23 (4.2)	19.76 (4.33)	0.002
AQLQ score, mean (SD) (V0)	4.58 (1.27)	5.34 (1.2)	0.092
Symptomatic patients (ACT < 20), *n* (%)	5 (38.5)	5 (10.9)	0.019
Exacerbations, mean (SD)	0.92 (1,12)	1.33 (1.63)	0.0621
Patients with an obstructive pattern (FEV_1_ < 80%), *n* (%)	6 (46.1)	15 (32.6)	0.368
Some allergic sensitization, *n* (%)	13 (100)	39 (90.7) *	0.254
Some clinically significant sensitization, *n* (%)	10 (76.9)	32 (69.6)	0.605
Total IgE, kU/L, mean (SD)	1007.93 (1416.04)	615.57 (735.25)	0.698
FeNO levels, ppb, mean (SD)	58.58 (60.44)	43.49 (46.20)	0.192
Serum periostin, pg/mL, mean (SD)	16,727 (14,098)	10,588 (11,635)	0.139
Sputum eosinophils, %, mean (SD)	2.61 (3.92)	2.52 (2.78)	0.469
Sputum eosinophils > 3%, *n* (%)	2 (22.2) ^†^	8 (36.4) ^‡^	0.445
Sputum neutrophils, %, mean (SD)	52.3(33.8)	51(32.8)	1
Patients with sputum neutrophils > 61%	4 (44.4) ^†^	10 (45.4) ^‡^	0.959
Blood eosinophil count, cells/µL, mean (SD)	0.53 (0.38)	0.6 (0.39)	0.330
Blood eosinophil count, %, mean (SD)	6.63 (5.04)	8.44 (4.69)	0.064
Sputum cellularity profile, *n* (%)			
Eosinophilic	2 (22.2) ^†^	7 (31.8) ^‡^	0.593
Neutrophilic	3 (33.3) ^†^	4 (17.2) ^‡^	0.360
Mixed	1 (11.1) ^†^	6 (27.3) ^‡^	0.329
Pauci-granulocytic	3 (33.3) ^†^	5 (22.7) ^‡^	0.540

SD: standard deviation; V0: initial visit; FEV_1_: forced expiratory value in one second; ACT: Asthma Control Test; AQLQ: Asthma Quality of Life Questionnaire; FeNO: fractional exhaled nitric oxide; * recorded in 43 patients; ^†^ recorded in 9 patients; ^‡^ recorded in 22 patients.

## Data Availability

Data supporting reported results are available from the corresponding author upon request.

## Supplementary Materials

The following supporting information can be downloaded at: https://www.mdpi.com/article/10.3390/jcm13237202/s1, Table S1: Correlation between eosinophils in sputum and FeNO, serum periostin, and blood eosinophils; Table S2: Inflammatory and immune-related variables in sputum and peripheral blood at 12 months of follow-up according to the level of asthma control; Table S3: Results of multiple logistic regression analysis of factors associated with uncontrolled asthma at 12 months of follow-up; Figure S1: ROC curve for regression model associated with uncontrolled asthma; Figure S2: Boxplot of the relationship between NCR^+^ILC3 cell populations and uncontrolled asthma at 12 months of follow-up, with higher values: (a) in patients without sensitization to tree pollen (left) and (b) in patients without sensitization to Parietaria pollen; Figure S3: Boxplot of the relationship between BMI and uncontrolled asthma after 12 months of follow-up in patients aged over 12 years.
