# Supplementary material for: Prognostic Factors in Severe Eosinophilic Asthma in a Pediatric Population: A Prospective Cohort Study in Spain
Source: J Clin Med. 2024 Nov 27;13(23):7202. doi: 10.3390/jcm13237202 (PMC11642421; doi:10.3390/jcm13237202)

Supplementary Material

Table S1. Inflammatory and immune-related variables in sputum and peripheral blood at 12 months of follow-up according to the level of asthma control

Variables	Never controlled asthma (n = 13)	Sometimes/always controlled asthma (n = 46)	*p* value
Sputum interleukins (IL), pg/m, mean (SD)			
IL-4	9.51 (8.26)	5.67 (9.08)	0.453
IL-5	2.62 (2.73)	3.16 (3.69)	0. 879
IL-8	5.81 (3.43)	3.27 (1.63)	0.297
IL-9	13.09 (10.54)	8.47 (5.20)	0.571
IL-13	9.16 (7.85)	4.06 (1.26)	0.456
IL-17	37.22 (26.76)	16.38 (7.65)	0.297
Blood interleukins (IL), pg/mL, mean (SD)			
IL-4	1.33 (1.58)	3.38 (4.97)	0.314
IL-5	1.11 (1.64)	1.26 (2.47)	0.945
IL-8	12.96 (5.29)	14.31 (16.06)	0.377
IL-9	2.14 (3.04)	3.91 (4.25)	0.069
IL-13	0.92 (1.50)	0.41 (0.77)	0.446
IL-17	1.60 (1.15)	1.62 (1.53)	0.531
Cell populations, mean (SD)			
Th1 effector, %	7.5 (5.58)	6.4 (3.58)	0.935
Th1 central memory, %	9 (2.71)	8.2 (3.1)	0.483
Th2 effector, %	3.06 (2.05)	2.42 (1.91)	0.383
Th2 central memory	6.29 (2.59)	5.82 (3.14)	0.295
Th17 effector, %	1.93 (0.84)	1.71 (0.81)	0.415
Th17 central memory, %	4.61 (2.08)	4.23 (1.81)	0.658
ILC1, ‰	0.19 (0.15)	0.17 (0.09)	0.889
ILC2, ‰	0.53 (0.24)	0.5 (0.28)	0.593
NCR^{-}ILC3, ‰	0.27 (0.13)	0.22 (0.14)	0.185
NCR^{+}ILC3, ‰	0.01 (0.01)	0.004 (0.005)	0.090

SD: standard deviation; NCR: natural cytotoxic receptor.

Table S2. Correlation between eosinophils in sputum and FeNO, serum periostin and blood eosinophils

Eosinophils in sputum	FeNO	Serum periostin pg/mL	Blood eosinophils %	Blood eosinophils cells/μL
Spearman rank correlation coefficient	0.030	0.164	0.181	0.058
P value	0.879	0.377	0.376	0.756

Table S3. Results of multiple logistic regression analysis of factors associated with uncontrolled asthma at 12 months of follow-up

Variables	Odds ratio	Confidence interval 95%	*P* value	Odds ratio	P5% confidence interval	*P* value
Asthma duration	1.226	1.010-1.489	0.04	1.263	1.006-1.587	0.04
ACT score at baseline	0.800	0.686-0.933	0.004	0.814	0.692-0.958	0.013
Serum IL-19 level	0.828	0.629-1.090	0.178			

Figure S1. ROC curve for regression model associated with uncontrolled asthma (AUC: 0.827, 95% CI 0.72-0.93; p < 0.000).

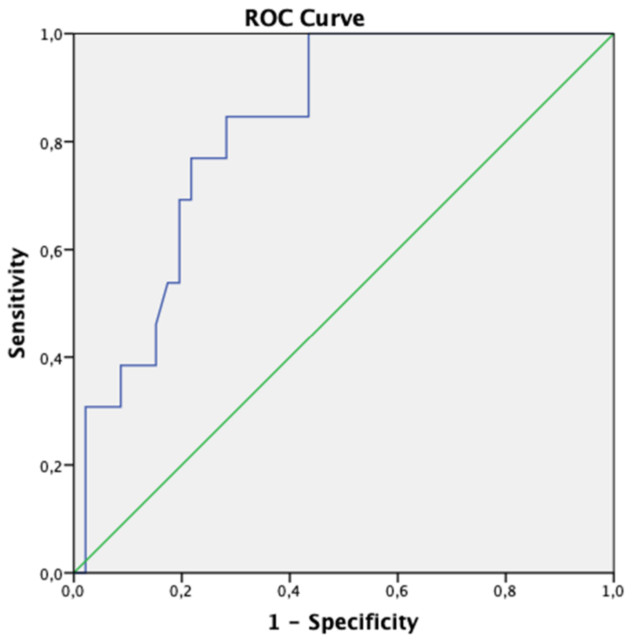

Figure S2. Boxplot of the relationship between NCR+ILC3 cell populations and uncontrolled asthma at 12 months of follow-up, with higher values: a) in patients without sensitization to tree pollen (left) and b) in patients without sensitization to Parietaria pollen.

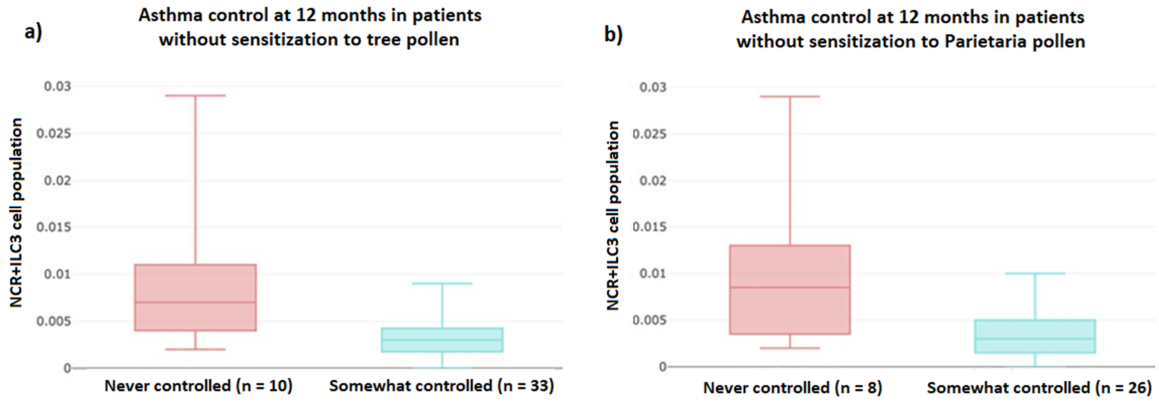

Figure S3. Boxplot of the relationship between BMI and uncontrolled asthma after 12 months of follow-up in patients aged over 12 years.

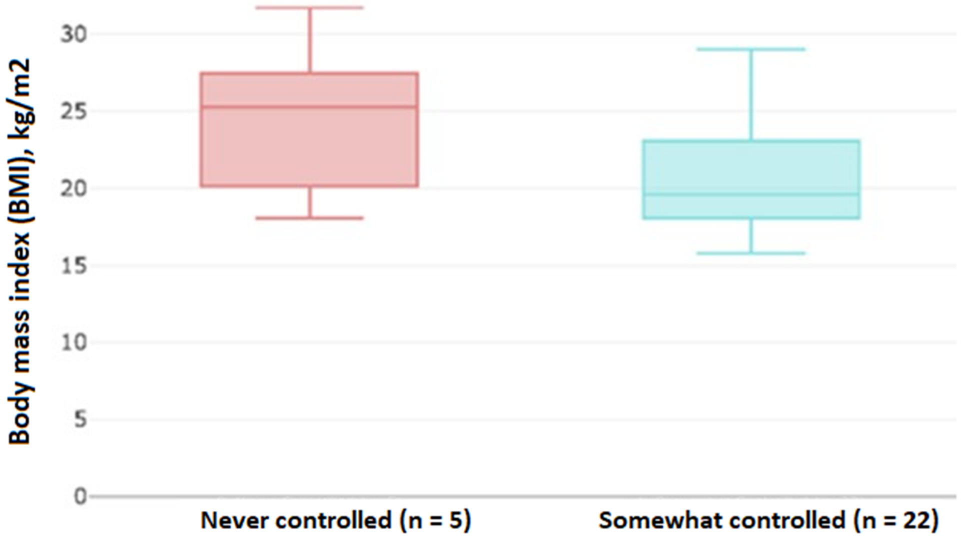

Supplement: Supplementary file 1 [file jcm-13-07202-s001.zip › jcm-3312432-supplementary.pdf]
